# Impact of early versus conventional kidney replacement therapy initiation in tumor lysis syndrome: a target trial emulation

**DOI:** 10.1186/s13613-025-01439-x

**Published:** 2025-04-04

**Authors:** Justine Serre, Guillaume Mulier, Charlotte Boud’hors, Marie Lemerle, Moustafa Abdel-Nabey, Corentin Orvain, Anis Chaba, Lucie Biard, Julien Demiselle, Lara Zafrani

**Affiliations:** 1 Department of Medical Intensive Care, Hôpital Saint-Louis, AP-HP, Paris, France; 2 Department of Biostatistics and Medical Information, AP-HP, Hôpital Saint-Louis, Université Paris Cité, Paris, France; 3 Department of Nephrology, Dialysis, Transplantation, CHU Angers, Angers, France; 4 Department of Medical Intensive Care, CHU Angers, Angers, France; 5 Department of Hematology, CHU Angers, Angers, France; 6 Federation hospitalo-universitaire « Grand Ouest against Leukemia », Nantes, France; 7 Inserm UMR 1307, CNRS UMR 6075, Nantes Université, Université d’Angers, Angers, CRCI2NA France; 8 Department of Medical Intensive Care, Nouvel Hôpital Civil, Strasbourg University Hospital, Strasbourg, France; 9 CRBS (Centre de Recherche en Biomédecine de Strasbourg), FMTS (Fédération de Médecine Translationnelle de Strasbourg), INSERM UMR 1260, Regenerative Nanomedicin, University of Strasbourg, Strasbourg, France; 10 INSERM UMR 944, Université Paris Cité, Paris, France

**Keywords:** Tumor lysis syndrome, Acute kidney injury, Kidney replacement therapy

## Abstract

**Background:**

In the context of tumor lysis syndrome (TLS), the optimal timing and criteria for initiating kidney replacement therapy (KRT) remain unclear. This study aims to assess the effect of initiating KRT at various phosphatemia thresholds on Major Adverse Kidney Events at day 30 (MAKE30).

**Methods and results:**

We retrospectively emulated a pragmatic clinical trial comparing the effect of KRT initiation at various phosphatemia thresholds *versus* a conventional approach during TLS on MAKE30. All consecutive patients admitted to the ICU at Saint-Louis University hospital in Paris and Angers University hospital between January 2007 and June 2020, presenting with laboratory TLS were included. The design criteria of a clinical trial were mimicked by using the cloning, censoring and weighting method. The primary outcome was the MAKE30 composite outcome, considering only KRT requirement between day 7 and day 30 for the dialysis criteria. We evaluated multiple phosphatemia thresholds to guide KRT initiation, ranging from 6.20 mg.dL^-1^ to 9.30 mg.dL^-1^. Among the initial population of 220 patients, 192 were included in the emulated trial (median age 60 years old, with non-Hodgkin Lymphoma and Acute Leukemia being the most frequent hematological malignancies). TLS-related AKI occurred in 140 patients, and 75 patients met the criteria for MAKE30. Regardless of the phosphate threshold considered, KRT initiation based on phosphate level was not associated with a significant difference in the MAKE30 rate. KRT requirement during the first 7 days (Odd Ratio [OR] 4.01 [1.65–4.86], *p* = 0.003) and non-renal SOFA (OR 1.39 per 1 point increment [1.25–1.57], *p* < 0.001) were identified as factors associated with MAKE30 (multivariable analysis).

**Conclusion:**

Our results do not support the strategy of KRT initiation based on a sole critical phosphatemia level in TLS patients.

**Supplementary Information:**

The online version contains supplementary material available at 10.1186/s13613-025-01439-x.

## Introduction

Tumor lysis syndrome (TLS) is a consequence of massive malignant cell destruction resulting in the release of large amounts of intracellular components into the systemic circulation. TLS is a life threatening complication occurring mostly in patients with hematological malignancies but can also occur in patients with solid tumors [1]. Laboratory TLS is defined by the presence of at least two of the following biological criteria: an elevation of uric acid, potassium levels or phosphate levels, or a reduction in calcium levels just before or within 7 days after chemotherapy [2]. Clinical TLS is defined by the onset of acute kidney injury (AKI), cardiac dysrhythmias or seizures [2]. In the context of TLS, the presence of AKI is associated with a poor prognosis, characterized by a lower rate of remission of the underlying malignancy [3] and an increased risk of mortality [4]. The pathophysiology of AKI is multifaceted and has yet incompletely been understood. To date, the predominant hypothesis is a crystal-induced nephropathy by calcium phosphate [5, 6], uric acid and xanthine precipitation in kidney tubules. Other recent studies have shown a pathogenic role of renal endothelial lesions induced by histone release during TLS [7]. The primary management approach for TLS involves extensive hydration with physiological saline and the administration of hypouricemic agents [8]. The role of kidney replacement therapy (KRT) in the context of TLS remains unclear and lacks well-defined parameters. Consensus on the criteria for initiating KRT includes situations of impending hyperkalaemia, prolonged oligoanuria, and fluid overload [9, 10]. There is ongoing debate on the potential advantages of early KRT initiation, with the goal of removing harmful intracellular compounds, especially for hyperphosphatemia, before crystal formation or endothelial injury [10]. As data focusing on KRT during TLS primarily comprises case series, further investigation is necessary.

Several clinical trials have investigated the optimal timing for initiating KRT in AKI [11–13]. However, it’s noteworthy that these studies excluded patients with TLS. To the best of our knowledge, there has been no exploration of the potential benefits of KRT specifically in the context of TLS. Additionally, no established cutoff for phosphatemia has been proposed to guide the initiation of KRT in TLS patients.

Ideally, addressing this question would require investigation through a prospective controlled trial. However, given the rarity of TLS, the feasibility of conducting such a trial is unlikely. Therefore, we adopted an innovative approach, namely to analyze the potential benefits of KRT initiated based on phosphatemia in patients with TLS, utilizing observational data in an emulated trial analysis.

## Methods

This retrospective multicenter cohort study was led in two university hospital Intensive care unit (ICU).

### Population

We included consecutive patients admitted to the ICU at Saint-Louis University hospital in Paris and Angers University hospital (France), between January 2007 and June 2020, all presenting with laboratory TLS. Patients were admitted to the ICU either from outside the hospital or transferred from hematology wards. In both institutions, senior hematologists and intensivists are available around the clock, seven days a week, collaboratively managing high-risk hematological patients. The ICU admission policies remained constant throughout the study period.

### Data collection

Patient characteristics, such as age, sex and medical history were extracted from medical records. Information pertaining to hematologic malignancy included details on the type of hematologic malignancy, treatments administered, and survival outcomes. Baseline creatinine levels, AKI KDIGO stage, initiation and duration of KRT in the ICU and kidney function recovery after discharge were collected. Biological data available for the patients were extracted from electronic medical records specifically including creatinine levels, urea, potassium levels, calcium levels, phosphatemia levels and lactate dehydrogenase (LDH). Blood tests were conducted every 6 h during ICU hospitalization.

### Definitions

Laboratory and clinical TLS were defined according to Cairo & Bishop criteria [2]. AKI was defined by an increase in serum creatinine and/or a decreased urine output according to KDIGO 2012 recommendations within 7 days after TLS diagnosis [14]. The classification of AKI stage was established prior to the initiation of the first KRT to prevent potential overclassification for patients undergoing dialysis for TLS without meeting the conventional criteria for KRT. The decision for KRT initiation, categorized as either “conventional approach” or “TLS specific” was determined by the attending clinician. In cases where relevant information was absent from the medical report, this categorization was made retrospectively. KRT initiation was considered “conventional” when one or more of the following severe laboratory abnormalities was observed : hyperkalaemia (K > 6 mEq.L-1), pH below 7.15 related to metabolic acidosis, or acute pulmonary edema due to fluid overload responsible for severe hypoxemia [11]^,^ [15]. The risks associated with severe hyperkalaemia, metabolic acidosis, and fluid overload unresponsive to diuretics are well-established and justify timely KRT initiation.

Glomerular filtration rate (eGFR) was calculated using the CKD Epidemiology Collaboration equation [16]. Baseline creatinine was considered in a time period of a maximum 1 year and a minimum of 7 days before TLS diagnosis. When baseline creatinine levels were unavailable and no pre-existing kidney disease was documented, an eGFR of 75 ml/min/1.73 m² was arbitrarily assigned, as recommended [17, 18]. Major adverse kidney events within 30 days (MAKE30) is a composite outcome of death, initiation of new KRT, and a decline in kidney function, defined as a 200% increase from basal serum creatinine. This assessment is performed 30 days after the diagnosis of TLS [19, 20]. Given that KRT might be employed exclusively for TLS treatment without typical AKI indications during the acute phase of TLS, our criteria for MAKE30’s KRT component focused on KRT instances occurring between day 7 and day 30 after TLS diagnosis. Vital status at 30 days was available for all the patients except for four cases without follow-up information after ICU discharge.

### TLS management

TLS management involved hematologists, nephrologists and intensive care physicians adhering to the guidelines outlined by Jones et al. [9]. Hydration and hypouricemic drugs were administered according to recommended practices. Treatment encompassed hydration with an approximate infusion of 3000 ml/day of saline, aiming to maintain a urine output of 100 ml/m^2^/h. The hydration prescription was adapted every 6 h based on clinical assessment. Rasburicase was administered at a dose of 0.2 mg/kg/day for a duration of 3 to 7 days, with the reinfusion rate adapted to uric acid levels. Allopurinol was administered to patients with suspected glucose-6-phosphate dehydrogenase (G6PD) deficiency. Given the absence of data on the specific indication and timing of KRT in the context of TLS, the decision to initiate and determine modalities of KRT was at the discretion of the attending physician.

### Study design and patient selection

This observational study emulated a pragmatic clinical trial comparing the effect of KRT initiation timing during TLS on MAKE30, either based on phosphatemia thresholds *versus* a conventional approach. We constructed a retrospective cohort using medical records of Saint-Louis hospital and Angers hospital. Our analysis included consecutive patients who met the following criteria during the study period: biological or clinical TLS, hospitalized in ICU, without indication of KRT at baseline as per the conventional approach and at least one available phosphatemia before KRT initiation. We defined baseline as the first biological sample of phosphatemia as the date of TLS diagnosis.

Supplementary file 1 outlines the protocol of such a trial.

Explicit emulation of a trial, and in particular aligning the start of follow-up with the assignment of treatment strategies, eliminates immortal time bias, selection/ survivor bias, and lead time bias, which can significantly affect observational studies. This approach is widely adopted in the ICU and nephrology settings and is a strong methodological option, particularly for studying the timing of KRT initiation, when a randomized trial is not feasible [21, 22].

### Treatment strategies

The question was whether the initiation of KRT based on a phosphatemia threshold was superior to the “conventional approach” to improve kidney-related morbidity and survival. Using this approach, we evaluated a range of phosphatemia thresholds to guide the timing of KRT initiation: thresholds investigated ranged from 6.20 mg.dL^− 1^ to 9.30 mg.dL^− 1^ with equally spaced intervals of 0.31 mg.dL-1 (corresponding to a range from 2.0 mmol.L-1 to 3.0 mmol.L^− 1^ with equally spaced intervals of 0.1 mmol.L^− 1^). Strategies of KRT initiation were defined in the first seven days after TLS as opposed to KRT after 7 days that was a criteria included in the MAKE score.

#### Primary outcome

the primary composite outcome was the MAKE at 30 days after TLS diagnosis, as defined above.

### Analytic methods

First, we cloned each patient twice for each threshold of phosphatemia. Given *N* analyzed patients, we therefore obtained a sample size *2 N* in the emulated controlled clinical trial for each phosphatemia threshold. For each patient, one clone was assigned to the strategy of initiating KRT based on phosphatemia crossing a prespecified threshold and the other clone was assigned to the conventional strategy of KRT initiation. In the phosphatemia strategy, KRT could be initiated up to 24 h after the phosphatemia measurement.

Clones were then censored when they deviated from their assigned strategy. Specifically, in the phosphatemia strategy, patients were censored (i) at the end of their follow-up or at seven days if no KRT had been initiated and the phosphatemia threshold had not been met, (ii) at the time of KRT when KRT had been initiated without the threshold being crossed, or (iii) 24 h after phosphatemia crossed the threshold and no KRT had been initiated. In the conventional strategy, patients were censored (i) at the end of their follow-up or at seven days if no KRT had been initiated and the KRT criteria not met, (ii) at the time of KRT when the phosphatemia threshold was crossed less than 24 h before KRT initiation. As a result, all patients are initially in both groups and they are censored during follow-up according the aforementioned rules. We used inverse probability of censoring weighting with probability of censoring estimated by Cox regression, to account for informative censoring introduced by this cloning step. More information on the censoring models are displayed in supplementary file 2, figures S1 and S2. We used stabilized weights truncated at 1st and 99th percentiles to avoid over-influent weights. Lastly, for each candidate phosphatemia threshold, the association between KRT strategy (phosphatemia vs. conventional) and MAKE30 was assessed by hazards ratios estimated by weighted Cox regression with robust variance.

Sensitivity analyses were performed with untruncated weights, and by adding interaction terms in the censoring model.

As a secondary analysis, we studied the association between MAKE30 and KRT requirement as per the conventional strategy in the seven days following the TLS diagnosis. Multivariable logistic regression was fitted adjusted on clinically relevant explanatory variables. Analyses were performed on the complete cases sample.

All tests were two-sided and *p*-values lower than 0.05 were considered significant. Analyses were performed on R platform, version 4.3.0.

## Results

**Fig. 1:**
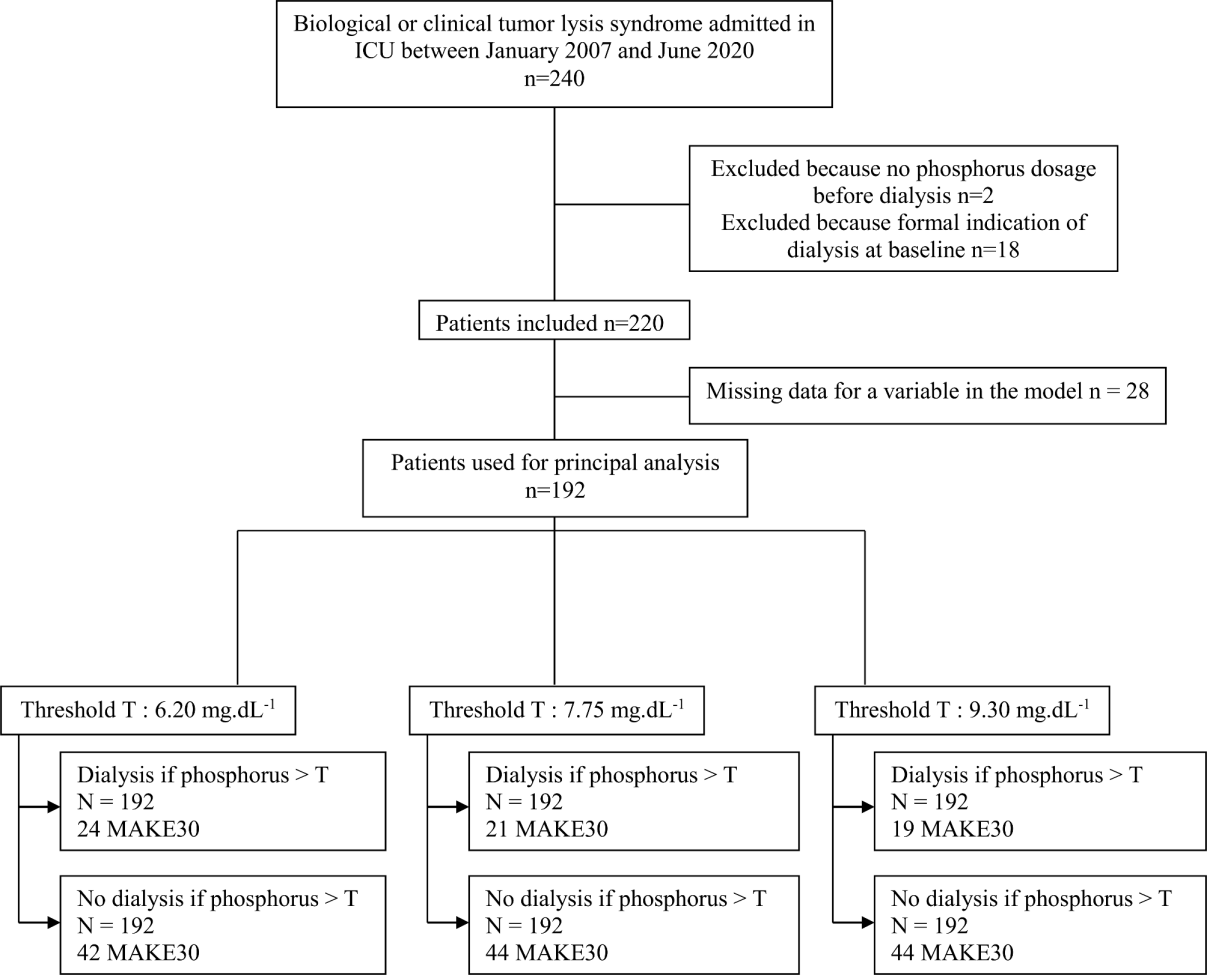
Flowchart of the emulated trial approach. The *n* = 192 patients in the analyzed set were cloned 1:1 in silico to obtain a 1:1 controlled sample. In the emulated intervention group, a sequence of phosphatemia values ranging from 6.20 to 9.30 mg/dL were evaluated to define the KRT decision threshold. For illustration purposes and legibility, the distribution of MAKE30 events per emulated group is reported for 3 phosphatemia thresholds (minimum, maximum and intermediate). KRT: Kidney replacement Therapy, MAKE: Major Adverse Kidney event

Among 240 eligible patients, 220 were included, with 192 of them participating in the emulated trial (see Fig. 1 for flowchart and reasons for exclusion). Of the 18 patients meeting “conventional” RRT criteria and thus excluded from the cohort for the purpose of the present analysis, the majority (*n* = 11, 61%) required KRT due to hyperkalaemia, while 4 patients (22%) required KRT for pulmonary overload associated with prolonged oliguria (> 72 h) and 3 (17%) for metabolic acidosis (pH < 7.15). Baseline characteristics of participants, including biological parameters at ICU admission, are presented in Table 1, according to KRT requirement.

**Table 1:** Baseline characteristics of patients according to KRT initiation

	All population (*n* = 220)	No KRT (*n* = 90)	KRT (*n* = 130)
***Baseline characteristics***			
Gender (F), n (%)	66 (30.0)	35 (38.9)	31 (23.8)
Age (Years), Med [IQR]	60 [48–69]	59 [45–69]	60 [50–68]
Baseline serum creatinine (mg.dL^− 1^), Med [IQR]	0.90 [0.76–1.03]	0.85 [0.75–1.01]	0.92 [0.78–1.03]
SOFA score, Med [IQR]	6 [3–9]	5 [3–7]	6 [3–9]
Modified SOFA score, Med [IQR]	4 [2–6]	4 [2–7]	4 [1–6]
***Underlying comorbidities***			
Hypertension, n (%)	63 (28.6)	23 (25.6)	40 (30.8)
Diabetes mellitus, n (%)	29 (13.2)	10 (11.1)	8 (14.3)
Chronic heart failure, n (%)	15 (6.9)	6 (6.8)	9 (6.9)
HIV positive, n (%)	29 (13.2)	7 (7.8)	22 (16.9)
Chronic Kidney Disease, n (%)	13 (5.9)	4 (4.4)	9 (6.9)
***Underlying malignancies***			
Acute Leukemia, n (%)	70 (31.8)	36 (40.0)	34 (26.2)
Non-Hodgkin lymphoma, n (%)	139 (63.2)	49 (54.4)	90 (69.2)
Multiple Myeloma, n (%)	3 (1.4)	2 (2.2)	1 (0.8)
Solid tumor, n (%)	3 (1.4)	2 (2.2)	1 (0.8)
Others, n (%)	5 (2.3)	1 (1.1)	4 (3.1)
***Risk factors for AKI***			
Nephrotoxic agents, n (%)	78 (35.5)	25 (27.8)	53 (40.8)
***TLS biochemical parameters at admission***			
Phosphate (mg.dL^− 1^), Med [IQR]	4.37 [3.41–5.80]	4.28 [3.35–5.22]	4.65 [3.41–6.20]
Uric Acid (mg.dL^− 1^), Med [IQR]	8.74 [5.63–12.36]	8.62 [5.74–11.61]	8.89 [5.62–13.10]
Potassium (mmol.L^− 1^), Med [IQR]	4.3 [3.9–4.8]	4.2 [3.6–4.6]	4.6 [4.1-5.0]
Calcium (mg.dL^− 1^), Med [IQR]	8.80 [8.00-9.60]	8.80 [8.00-9.60]	8.80 [8.00-9.60]
LDH (UI/L), Med [IQR]	2403 [1150–5082]	2318 [1157–5125]	2494 [1186–4798]
Serum creatinine (mg.dL^− 1^) Med [IQR]	1.38 [0.96–2.17]	1.12 [0.83–1.68]	1.60 [1.12–2.70]
**Disseminated Intravascular Coagulation**,** n (%)**	48 (21.8)	22 (24.4)	26 (20.0)

Data are presented as counts and percentages for categorical data and as median (Med) with interquartile range (IQR) for quantitative data

AKI: Acute Kidney Injury, HIV: Human Immunodeficiency Virus, LDH: Lactate DeHydrogenase, SOFA: Sequential Organ Failure Assessment, Modified SOFA: SOFA without kidney component, TLS: tumor lysis syndrome, KRT: Kidney Replacement Therapy

Patients were predominantly men, with a median age of 60 years old. Non-Hodgkin lymphoma and acute leukemia accounted for more than 90% of the underlying hematological malignancies causing TLS. Clinical TLS occurred in 140 patients, with kidney involvement being the main contributor, while neurological and hemodynamic involvement occurred in one and four patients respectively. Among the 140 (63.6%) patients with TLS-related AKI, 48 patients (21.8%) met the criteria for stage 3 of the KDIGO definition. Of note, chronic kidney disease was rare in our population.

Characteristics of TLS management and main outcomes are reported in Table 2.

**Table 2:** Tumor lysis management and main outcomes according to KRT initiation

	All population (*n* = 220)	No KRT (*n* = 90)	KRT (*n* = 130)
***Treatment of TLS***			
Allopurinol, n (%)$	77/217 (35.5)	32/89 (36.0)	45/128 (35.2)
Rasburicase, n (%)*	155/219 (70.8)	69 (76.7)	86/129 (66.7)
***TLS outcome***			
MAKE30, n (%)$	75/217 (34.6)	20/88 (22.7)	55/129 (42.6)
- Mortality criteria, n (%)$	61/217 (28.1)	19/88 (21.6)	42/129 (32.6)
- Kidney Replacement Therapy criteria, n (%)$	19/217 (8.8)	-	19/129 (14.7)
- Serum creatinine criteria, n (%)$	8/217 (3.7)	1/88 (1.1)	7/129 (5.4)
AKI, n (%)	140 (63.6)	31 (34.4)	109 (83.8)
- KDIGO 1, n (%)	52 (23.6)	18 (20.0)	34 (26.2)
- KDIGO 2, n (%)	40 (18.2)	8 (8.9)	32 (24.6)
- KDIGO 3, n (%)	48 (21.8)	5 (5.6)	43 (33.1)

Data are presented as counts and percentages for categorical data

$ information was unavailable in 3 patients, * information was unavailable in 1 patients, AKI: Acute Kidney Injury, KDIGO: Kidney Disease Improving Global Outcomes, MAKE30: Major Adverse Kidney Events at day 30, TLS: Tumor Lysis Syndrome. KRT: Kidney Replacement Therapy

The primary outcome was available in 217 patients. Seventy-five patients fulfilled criteria for MAKE30.

Next, using an emulated trial with the above-defined method of cloning, censoring and weighting, we assessed whether a strategy of initiation of KRT according to various phosphatemia thresholds, compared to a conventional strategy, affected the main outcome in the 192 patients with available data for the models.

**Fig. 2:**
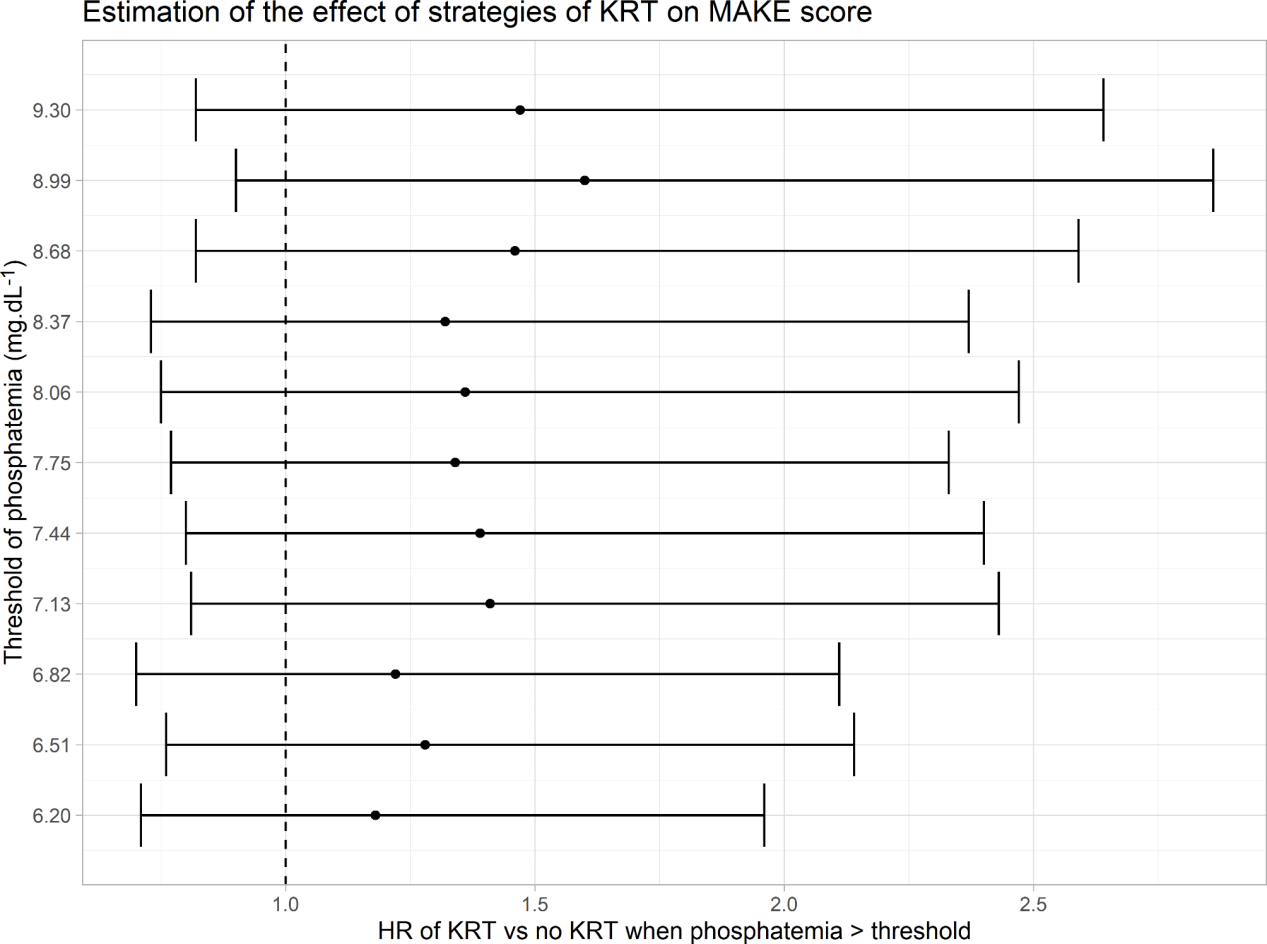
Results from principal analysis on MAKE score relative to the threshold of phosphatemia with truncated stabilized weights. It depicts the hazard ratio (strategy KRT vs. no KRT when phosphatemia exceeds the threshold) estimated by weighted Cox regression. KRT : Kidney replacement Therapy, MAKE: Major Adverse Kidney event

**Fig. 3:**
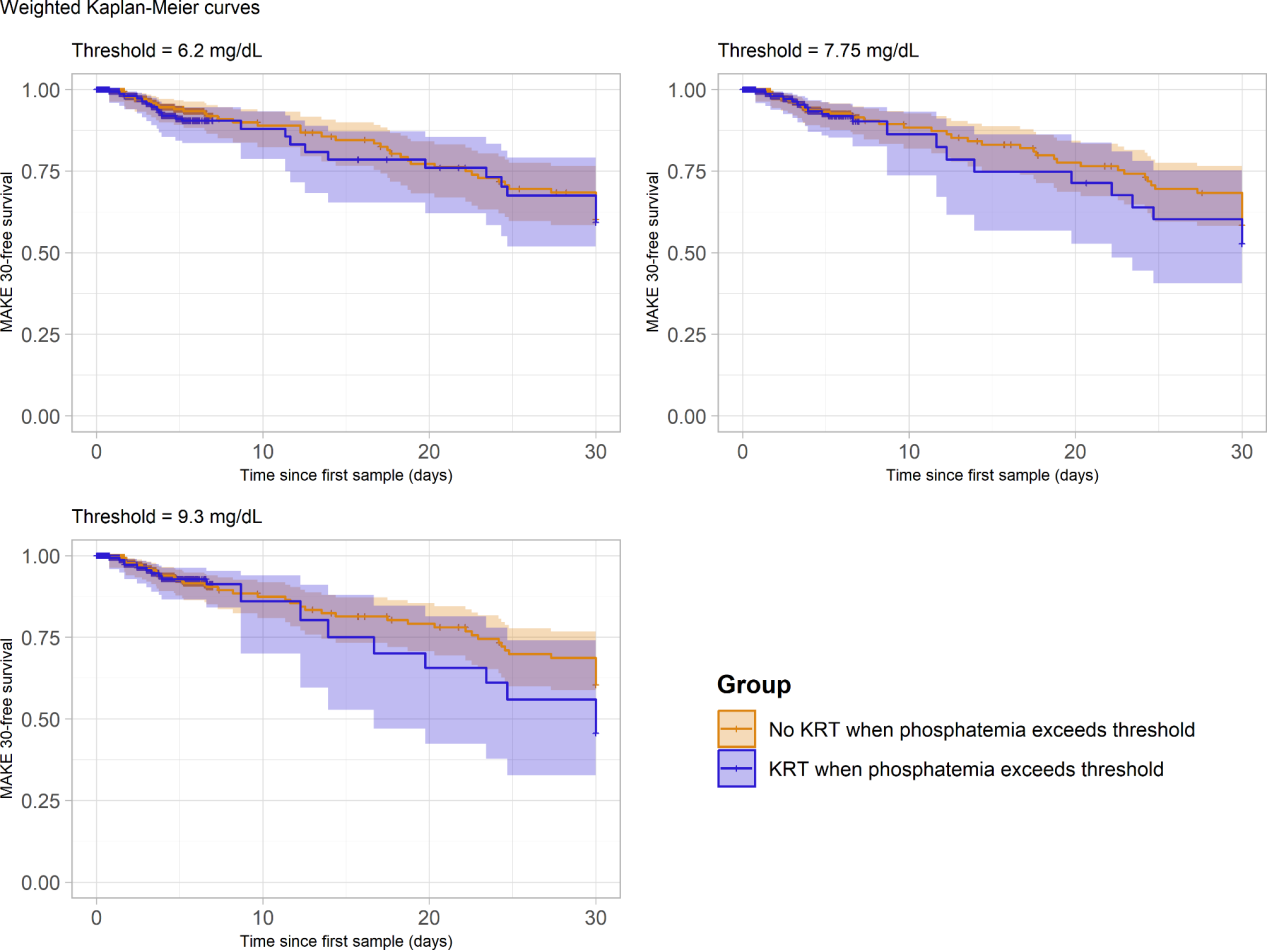
Weighted Kaplan-Meier curves of the MAKE30-free survival since first phosphate sampling according to KRT-decision group, using the emulated trial datasets. In the emulated intervention group, a sequence of phosphatemia values ranging from 6.20 to 9.30 mg/dL were evaluated to define the KRT decision threshold; for illustration purposes and legibility, the MAKE30-free survival by emulated group is reported for 3 phosphatemia thresholds only, across this range (minimum, maximum and intermediate)

As presented in Fig. 2, whatever the chosen phosphate threshold, the phosphatemia strategy for KRT initiation was not associated with a significant difference in MAKE30 as compared to the conventional approach. Kaplan Meier curves for three different values of phosphate threshold (Fig. 3) are reported to illustrate this finding, which was confirmed in sensitivity analysis (see Supplementary file 3, Figures S3, S4, S5).

Finally, to evaluate factors associated with MAKE-30, we performed univariate and multivariable analysis (Supplemental Tables 1 and Table 3, respectively). In multivariable analysis, KRT requirement during the first 7 days (Odd Ratio [OR] 4.01 [1.65–4.86], *p* = 0.003) and non-renal SOFA (OR 1.39 per 1 point increment [1.25–1.57], *p* < 0.001) were independently associated with MAKE30.

**Table 3:** Multivariable analysis of factors associated with MAKE30

Variable	OR	*P* value
No KRT at day 7	1 (Reference)	
KRT during the first 7 days	4.01 [1.65;4.86]	0.003
Non Hodgkin lymphoma	1 (Reference)	
Acute leukemia	1.19 [0.52;2.69]	0.682
Other	2.08 [0.47;9.75]	0.337
Non renal SOFA (by 1 point-increment)	1.39 [1.25;1.57]	< 0.001
LDH at admission (by 1000 UI/L increment)	0.99 [0.91;1.08]	0.884
Clinical tumor lysis syndrome	1.79 [0.72;4.65]	0.219

KRT: Kidney replacement therapy, LDH: Lactate DeHydrogenase, SOFA: Sequential Organ Failure Assessment

Among patients requiring KRT, dialysis catheter-related complications occurred in 7 (5.5%) patients with 3 catheter-related bloodstream infections and 4 cases of catheter-related bleeding at the puncture site requiring red blood cell transfusion.

## Discussion

In this study, we investigated the effectiveness of a preemptive KRT strategy based on phosphate levels during TLS with regard to a composite outcome encompassing kidney recovery and mortality. Despite testing various phosphate thresholds, an early KRT initiation strategy based on phosphatemia was not associated with improved patient’s outcomes.

There is a lack of studies addressing the indications and optimal timing of KRT during TLS. Guidelines by Jones et al. [9] recommend KRT for TLS patients exhibiting intractable fluid overload, hyperkalaemia, hyperuricemia, hyperphosphatemia, or hypocalcemia. If KRT is exceptionally required in the rasburicase era for hyperuricemia, fluid overload and hyperkalaemia remained classical emergency situations. Hyperphosphatemia as a sole criteria for beginning KRT remains a matter of debate. Hyperphosphatemia is an early marker for AKI in the setting of TLS [23], and some propose a phosphatemia threshold of 2 mmol/L to initiate KRT, even in the absence of AKI [10]. Prophylactic use of KRT (using a combination of Continuous Venovenous Hemofiltration (CVVH) with prephase chemotherapy) has also been proposed by some expert teams in adults and children, for patients with spontaneous TLS or at high risk of developing TLS [24, 25]. However, these suggestions are based on retrospective analyses of a small number of patients, precluding any firm conclusions regarding the efficacy and safety of this strategy and the optimal timing for initiating KRT in TLS-induced AKI remains uncertain [9]. Hence, the low level of recommendation on KRT indications and timing during TLS mirrors the absence of available randomized studies.

Importantly, existing evidence from general ICU settings indicates no benefit from early KRT during AKI. Three published large multicenter randomized control studies that compared early and delayed strategies of KRT [11, 13, 26] in critically ill patients did not found any benefit of an early strategy when compared to a delayed strategy, in terms of mortality. However, patients with TLS have been excluded from these studies [11].

In contrast to the broader ICU context, TLS presents a unique scenario where KRT has been suggested as an effective strategy to mitigate kidney structural damage caused by the release of high phosphorus levels from malignant cells. The levels of phosphorus in malignant cells can be up to four times higher than those found in normal cells. The swift release of these stores can lead to hyperphosphatemia, precipitating with calcium into the kidney tubules. This process induces calcium phosphate nephropathy, subsequently causing AKI, which further exacerbates hyperphosphatemia, thus creating a vicious cycle [5, 27]. Many physicians facing TLS commence KRT well before the development of any conventional indications for KRT. However, despite the plausible pathophysiological rationale, there is a lack of clinical evidence supporting the preemptive use of KRT in TLS.

The challenges associated with conducting a randomized study on KRT initiation during TLS are manifold due to the rarity of the disease, requiring close coordination among hematologists, oncologists, nephrologists, and intensivists. Additionally, logistical constraints, such as the need for central venous catheter insertion and ICU availability, further complicate such endeavors. Furthermore, retrospective studies face inherent difficulties in interpreting the timing of dialysis initiation, including concerns about immortal time, selection/survivor biases, and lead time biases. Our study design, incorporating cloning, censoring, and weighting, aims to mitigate these biases by emulating a target trial and aligning eligibility and treatment strategies at baseline [21]. Such an approach ensures to take into account time-varying confounders and immortal-time bias [28], and appears relevant in the setting of clinical research in the field of intensive care and nephrology research, allowing us to test our hypothesis for various phosphatemia thresholds for the timing of KRT.

However, this statistical approach suffers from limitations: it is an observational study, as opposed to a prospective controlled assessment in a randomized trial. Due to the observational design, we could only adjust for measured confounders. Thus, there may still be unmeasured confounding factors that could bias the results, such as confounding by indication, meaning unmeasured factors associated with patient’s risk in relation to KRT initiation. Moreover, we could not perform the emulated trial analysis using thresholds outside the range of 6.20 mg.dL^− 1^ to 9.30 mg.dL^− 1^ as more extreme thresholds were directly linked to KRT status with little to no variability (e.g. above 9.30 mg.dL^− 1^ nearly all patients had KRT).

Furthermore, the study involved patients from two specialized centers, with high expertise in the care of patients at high risk of TLS (hydration management, early rasburicase use for example), and, consequently, whether our findings can be generalized to other settings remain uncertain.

Two potential interpretations arise from our study findings:

Firstly, early KRT might not confer benefits in TLS patients. KRT might be ineffective in protecting the kidney from the release of harmful components by malignant cells during TLS. Moreover, the introduction of early KRT could potentially result in adverse effects, notably catheter-related complications, as illustrated in this work, especially in thrombocytopenic and immunosuppressed patients. Furthermore, KRT in cancer patients may be associated with modifications in the effectively received doses of chemotherapy regimens, potentially dampening the anti-tumoral effect of the drug, reducing the likelihood of achieving complete remission of the underlying malignancy. This is due to the fact that the pharmacokinetics of chemotherapeutic drugs in high-grade hematological malignancies in patients undergoing KRT are not well known.

Secondly, phosphate may not be an ideal marker for guiding KRT strategies in TLS, as other mechanisms, such as crystal-independent pathways involving extracellular histones, damage-associated molecular patterns, and pro-inflammatory cytokines, could contribute to AKI in TLS [29, 30]. Indeed, if AKI can be due to crystal dependent mechanisms during TLS, including calcium-phosphate nephropathy, Arnaud et al. showed that calcium-phosphate crystals are exceptional in TLS patients, since the guidelines do not recommend urine alkalinization. Moreover, they showed that TLS patients release huge amounts of extracellular histones into the circulation that may exert cytotoxic effects on kidney endothelial cells [30].

Another challenge in managing patients with TLS involves selecting the appropriate KRT modality—intermittent versus continuous. Some authors predominantly advocate for CVVH in TLS cases [25, 31, 32], while others favor daily intermittent hemodialysis [33]. Considering the continuous release of intracellular content from lysing tumor cells, continuous modalities are sometimes favored over intermittent hemodialysis to mitigate the risk of “rebound” hyperkalaemia or hyperphosphatemia. On the other hand, conventional hemodialysis demonstrates a more effective clearance of potassium and chemotherapy can be administered between two sessions, mitigating the risk of underdosing the treatment. In our study, we reported a consistent strategy with hemodialysis as the first modality of KRT in a large majority of included patients, which precluded us from drawing any firm conclusions about the best modality to use in TLS patients.

In conclusion, our results do not support the strategy of KRT initiation based on a sole critical phosphatemia level in TLS patients. Whether due to potential deleterious effects of early KRT or the inadequacy of serum phosphate as a relevant clinical marker for kidney associated outcomes in this setting, further investigations are warranted to refine KRT strategies in TLS.

## Electronic supplementary material

Below is the link to the electronic supplementary material.

Supplementary Material 1

Supplementary Material 2

Supplementary Material 3

## Data Availability

The datasets used and/or analyzed during the current study are available from the corresponding author on reasonable request.

## Abbreviations

TLS: Tumor lysis syndrome
AKI: Acute kidney injury
ICU: Intensive care unit
KRT: kidney replacement therapy
GFR: Glomerular filtration rate
MAKE30: Major adverse kidney events within 30 days
G6PD: glucose-6-phosphate dehydrogenase
CVVH: Continuous venovenous hemofiltration
SOFA: Sequential organ failure assessment

## References

[1] Mirrakhimov AE, Ali AM, Khan M, Barbaryan A. Tumor lysis syndrome in solid tumors: an up to date review of the literature. Rare Tumors. 2014;6:68–76. 10.4081/rt.2014.5389 PMC4083673 25002953

[2] Cairo MS, Bishop M. Tumour lysis syndrome: new therapeutic strategies and classification. Br J Haematol. 2004;127:3–11. 15384972 10.1111/j.1365-2141.2004.05094.x

[3] Canet E, Zafrani L, Lambert J, Thieblemont C, Galicier L, Schnell D, et al. Acute kidney Injury in patients with newly diagnosed high-Grade Hematological malignancies: Impact on Remission and Survival. PLoS ONE. 2013;8:e55870. 23457485 10.1371/journal.pone.0055870 PMC3573047

[4] Darmon M, Guichard I, Vincent F, Schlemmer B, Azoulay E. Prognostic significance of acute renal injury in acute tumor lysis syndrome. Leuk Lymphoma. 2010;51:221–7. 20001238 10.3109/10428190903456959

[5] Kanfer A, Richet G, Roland J, Chatelet F. Extreme hyperphosphataemia causing acute anuric nephrocalcinosis in lymphosarcoma. Br Med J. 1979;1:1320–1. 582154 10.1136/bmj.1.6174.1320-a PMC1599585

[6] Boles JM, Dutel JL, Briere J, Mialon P, Robasckiewicz M, Garre M, et al. Acute renal failure caused by extreme hyperphosphatemia after chemotherapy of an acute lymphoblastic leukemia. Cancer. 1984;53:2425–9. 6585264 10.1002/1097-0142(19840601)53:11<2425::aid-cncr2820531111>3.0.co;2-r

[7] Arnaud M, Loiselle M, Vaganay C, Pons S, Letavernier E, Demonchy J, et al. Tumor lysis syndrome and AKI: beyond Crystal mechanisms. J Am Soc Nephrol. 2022;33:1154. 35523579 10.1681/ASN.2021070997 PMC9161807

[8] Cairo MS, Coiffier B, Reiter A, Younes A. Recommendations for the evaluation of risk and prophylaxis of tumour lysis syndrome (TLS) in adults and children with malignant diseases: an expert TLS panel consensus. Br J Haematol. 2010;149:578–86. 20331465 10.1111/j.1365-2141.2010.08143.x

[9] Jones GL, Will A, Jackson GH, Webb NJA, Rule S. British Committee for Standards in Haematology. Guidelines for the management of tumour lysis syndrome in adults and children with haematological malignancies on behalf of the British Committee for Standards in Haematology. Br J Haematol. 2015;169:661–71. 25876990 10.1111/bjh.13403

[10] Coiffier B, Altman A, Pui C-H, Younes A, Cairo MS. Guidelines for the management of Pediatric and adult tumor lysis syndrome: an evidence-based review. JCO. 2008;26:2767–78. 10.1200/JCO.2007.15.0177 18509186

[11] Gaudry S, Hajage D, Schortgen F, Martin-Lefevre L, Pons B, Boulet E, et al. Initiation strategies for renal-replacement therapy in the Intensive Care Unit. N Engl J Med. 2016;375:122–33. 27181456 10.1056/NEJMoa1603017

[12] Wald R, Bagshaw SM, Investigators STARRT-AKI. Timing of initiation of renal-replacement therapy in Acute kidney Injury. Reply. N Engl J Med. 2020;383:1797–8. 33113308 10.1056/NEJMc2027489

[13] Barbar SD, Clere-Jehl R, Bourredjem A, Hernu R, Montini F, Bruyère R, et al. Timing of renal-replacement therapy in patients with acute kidney Injury and Sepsis. N Engl J Med. 2018;379:1431–42. 30304656 10.1056/NEJMoa1803213

[14] Acute Kidney Injury (AKI). – KDIGO. [cited 2021 Jun 22]. Available from: https://kdigo.org/guidelines/acute-kidney-injury/

[15] Ostermann M, Bellomo R, Burdmann EA, Doi K, Endre ZH, Goldstein SL et al. Controversies in acute kidney injury: conclusions from a Kidney Disease: Improving Global Outcomes (KDIGO) Conference. Kidney International. 2020;98:294–309. 10.1016/j.kint.2020.04.020 PMC8481001 32709292

[16] Inker LA, Eneanya ND, Coresh J, Tighiouart H, Wang D, Sang Y, et al. New Creatinine- and cystatin C-Based equations to Estimate GFR without Race. N Engl J Med. 2021;385:1737–49. 34554658 10.1056/NEJMoa2102953 PMC8822996

[17] Bellomo R, Ronco C, Kellum JA, Mehta RL, Palevsky P, Acute Dialysis Quality Initiative workgroup. Acute renal failure - definition, outcome measures, animal models, fluid therapy and information technology needs: the Second International Consensus Conference of the Acute Dialysis Quality Initiative (ADQI) Group. Crit Care. 2004;8:R204-212. 10.1186/cc2872 PMC522841 15312219

[18] Khwaja A. KDIGO clinical practice guidelines for acute kidney injury. Nephron Clin Pract. 2012;120:c179–184. 22890468 10.1159/000339789

[19] Semler MW, Rice TW, Shaw AD, Siew ED, Self WH, Kumar AB, et al. Identification of Major Adverse Kidney Events within the Electronic Health Record. J Med Syst. 2016;40:167. 27234478 10.1007/s10916-016-0528-z PMC5791539

[20] Kellum JA, Zarbock A, Nadim MK. What endpoints should be used for clinical studies in acute kidney injury? Intensive Care Med. 2017;43:901–3. 28255614 10.1007/s00134-017-4732-1

[21] Fu EL, Evans M, Carrero J-J, Putter H, Clase CM, Caskey FJ et al. Timing of dialysis initiation to reduce mortality and cardiovascular events in advanced chronic kidney disease: nationwide cohort study. BMJ. 2021; BMJ . 2021 Nov 29:375:e066306 10.1136/bmj-2021-066306 PMC8628190 34844936

[22] Mellado-Artigas R, Borrat X, Ferreyro BL, Yarnell C, Hao S, Wanis KN, et al. Effect of immediate initiation of invasive ventilation on mortality in acute hypoxemic respiratory failure: a target trial emulation. Crit Care. 2024;28:157. 38730306 10.1186/s13054-024-04926-y PMC11088053

[23] Lemerle M, Schmidt A, Thepot-Seegers V, Kouatchet A, Moal V, Raimbault M, et al. Serum phosphate level and its kinetic as an early marker of acute kidney injury in tumor lysis syndrome. J Nephrol. 2022;35:1627–36. 35107777 10.1007/s40620-022-01263-7

[24] Saccente SL, Kohaut EC, Berkow RL. Prevention of tumor lysis syndrome using continuous veno-venous hemofiltration. Pediatr Nephrol. 1995;9:569–73. 8580012 10.1007/BF00860936

[25] Choi KA, Lee JE, Kim Y-G, Kim DJ, Kim K, Ko YH, et al. Efficacy of continuous venovenous hemofiltration with chemotherapy in patients with Burkitt lymphoma and leukemia at high risk of tumor lysis syndrome. Ann Hematol. 2009;88:639–45. 19030857 10.1007/s00277-008-0642-1

[26] Investigators STARRT-AKI, Canadian Critical Care Trials Group, Australian and New Zealand Intensive Care Society Clinical Trials Group, United Kingdom Critical Care Research Group, Canadian Nephrology Trials Network, Irish Critical Care Trials Group. Timing of initiation of renal-replacement therapy in Acute kidney Injury. N Engl J Med. 2020;383:240–51. 32668114 10.1056/NEJMoa2000741

[27] Howard SC, Jones DP, Pui C-H. The tumor lysis syndrome. N Engl J Med. 2011;364:1844–54. 21561350 10.1056/NEJMra0904569 PMC3437249

[28] Cain LE, Robins JM, Lanoy E, Logan R, Costagliola D, Hernán MA. When to Start Treatment? A Systematic Approach to the Comparison of Dynamic Regimes Using Observational Data. The International Journal of Biostatistics. 2010 [cited 2023 Jan 5];6. Available from: https://www.degruyter.com/document/doi/10.2202/1557-4679.1212/html 10.2202/1557-4679.1212 PMC3406513 21972433

[29] Joseph A, Zafrani L. How I treat tumor lysis syndrome. Clin J Am Soc Nephrol. 2023;18:1634–6. 37788006 10.2215/CJN.0000000000000331 PMC10723907

[30] Arnaud M, Loiselle M, Vaganay C, Pons S, Letavernier E, Demonchy J, et al. Tumor lysis syndrome and AKI: beyond Crystal mechanisms. JASN. 2022;33:1154–71. 35523579 10.1681/ASN.2021070997 PMC9161807

[31] Anderson A, Shoulders L, James V, Ashcraft E, Cheng C, Ribeiro R, et al. Benefit of continuous kidney replacement therapy for managing tumor lysis syndrome in children with hematologic malignancies. Front Oncol. 2023;13:1234677. 37664024 10.3389/fonc.2023.1234677 PMC10471890

[32] Agha-Razii M, Amyot SL, Pichette V, Cardinal J, Ouimet D, Leblanc M. Continuous veno-venous hemodiafiltration for the treatment of spontaneous tumor lysis syndrome complicated by acute renal failure and severe hyperuricemia. Clin Nephrol. 2000;54:59–63. 10939758

[33] Abu-Alfa AK, Younes A. Tumor lysis syndrome and acute kidney injury: evaluation, prevention, and management. Am J Kidney Dis. 2010;55:S1–13. quiz S14-19. 20420966 10.1053/j.ajkd.2009.10.056

