# Supplementary material for: Impact of early versus conventional kidney replacement therapy initiation in tumor lysis syndrome: a target trial emulation
Source: Ann Intensive Care. 2025 Apr 4;15:49. doi: 10.1186/s13613-025-01439-x (PMC11968619; doi:10.1186/s13613-025-01439-x)
Supplement: Supplementary file 1 — Supplementary Material 1 [file 13613_2025_1439_MOESM1_ESM.docx]

**Supplementary file 1: Target trial emulated**

| Component | Target trial. | Emulated trial. |
| --- | --- | --- |
| Design | Randomized, prospective, multicentric, open-label trial. Interim analysis with decision rules of futility and efficacy. | Retrospective cohort of patients admitted in ICU within 48 hours after TLS. |
| Objective | Evaluate a strategy of early KRT based on phosphatemia thresholds vs conventional strategy on the MAKE 30 score. | Evaluate a strategy of early KRT based on phosphatemia thresholds vs conventional strategy on the MAKE 30 score. |
| Eligibility | - Adults | Same criteria. |
|  | - Consent to participate |  |
|  | - Tumor lysis syndrome defined by Cairo and Bishop in 2004 (biological and clinical criteria); |  |
|  | - No indication to emergency KRT among serum potassium concentration over 6 mmol.L^-1^, pH below 7.15 related to metabolic acidosis, , and acute pulmonary edema due to fluid overload responsible for severe hypoxemia:; |  |
|  | - No end stage chronic kidney disease (GFR<15 ml/min); |  |
|  | - No dialysis; |  |
|  | - No pregnancy.  - ICU admission within 48h of TLS diagnosis. |  |
| Treatment | KRT in the 7 days after TLS defined as KRT initiation when phosphatemia is greater than a threshold T, and conventional if KRT is initiated only if patients has at least one criteria among: serum potassium concentration over 6 mmol.L^-1^, pH below 7.15 related to metabolic acidosis, and acute pulmonary edema due to fluid overload responsible for severe hypoxemia. | KRT initiation if phosphatemia exceeds threshold T vs no KRT if phosphatemia exceeds that threshold. |
| Treatment assignment | Stratified randomization on center. | Retrospective cohort on Saint-Louis and Angers hospital data. |
| Endpoints | MAKE 30 and hospital mortality. | Time to event MAKE within 30 days. |
| Type of endpoint | Binary. | Survival. |
| Adjustment | No adjustment. | Adjustment on confounding factors in the censoring model and on residual imbalance in analysis model. |
| Causal contrast | Intend to treat. | Per protocol. |
| Estimator | Odds Ratio. | Hazard Ratio. |

ICU: Intensive care unit, KRT: Kidney replacement therapy, MAKE: Major adverse kidney event, TLS: Tumor lysis syndrome.
