# Supplementary material for: Impact of early versus conventional kidney replacement therapy initiation in tumor lysis syndrome: a target trial emulation
Source: Ann Intensive Care. 2025 Apr 4;15:49. doi: 10.1186/s13613-025-01439-x (PMC11968619; doi:10.1186/s13613-025-01439-x)
Supplement: Supplementary file 2 — Supplementary Material 2 [file 13613_2025_1439_MOESM2_ESM.docx]

**Supplementary file 2: Censoring model**

Separate models for patients’ censoring processes were estimated in each strategy (phosphatemia vs conventional) of each patient clone.

Linear predictor of the Cox regression model for patients’censoring in the conventional strategy (KRT initiation based on conventional criteria): $\beta_{1}\text{SLS}+\beta_{2}\text{Sex}+\beta_{3}\log\left( \text{Phosphatemia before 1.2 days} \right)+\beta_{4}log\left( \text{Phosphatemia at 1.2 days or more} \right)+\beta_{5}\text{High blood pressure}+\beta_{6}\text{Diabete}+\beta_{7}\text{Non renal SOFA}+\beta_{8}\text{Uricemia }\text{at admission}+\beta_{9}\text{Phosphatemia at admission}+\beta_{10}\text{Calcemia at admission}+\beta_{11}\text{Creatininemia at admission}$.

Linear predictor of the Cox regression model for patient’s censoring in phosphatemia strategy (KRT initiation when phosphatemia exceeds a pre-specified threshold): $\beta_{1}\text{SLS}+\beta_{2}\text{Sex}\text{+}\beta_{3}\text{Phosphatemia}+\beta_{4}\text{High blood pressure}+\beta_{5}\text{Diabete}\text{s}+\beta_{6}\text{Non renal SOFA}+\beta_{7}\text{Uricemia at admission}+\beta_{8}\text{Phosphatemia at admission before 1.2 days}+\beta_{9}\text{Phosphatemia at admission at 1.2 days or more}+\beta_{10}\text{Calcemia at admission}+\beta_{11}\log\left( \text{Creatininemia at admission before 1.2 days} \right)+\beta_{12}\log\left( \text{Creatininemia at admission at 1.2 days or more} \right)$.

**Figure S1: Censoring weights on the original dataset relative to the threshold of phosphatemia (stabilized weights truncated at 2% in bilateral). The red line is the LOESS curve.**


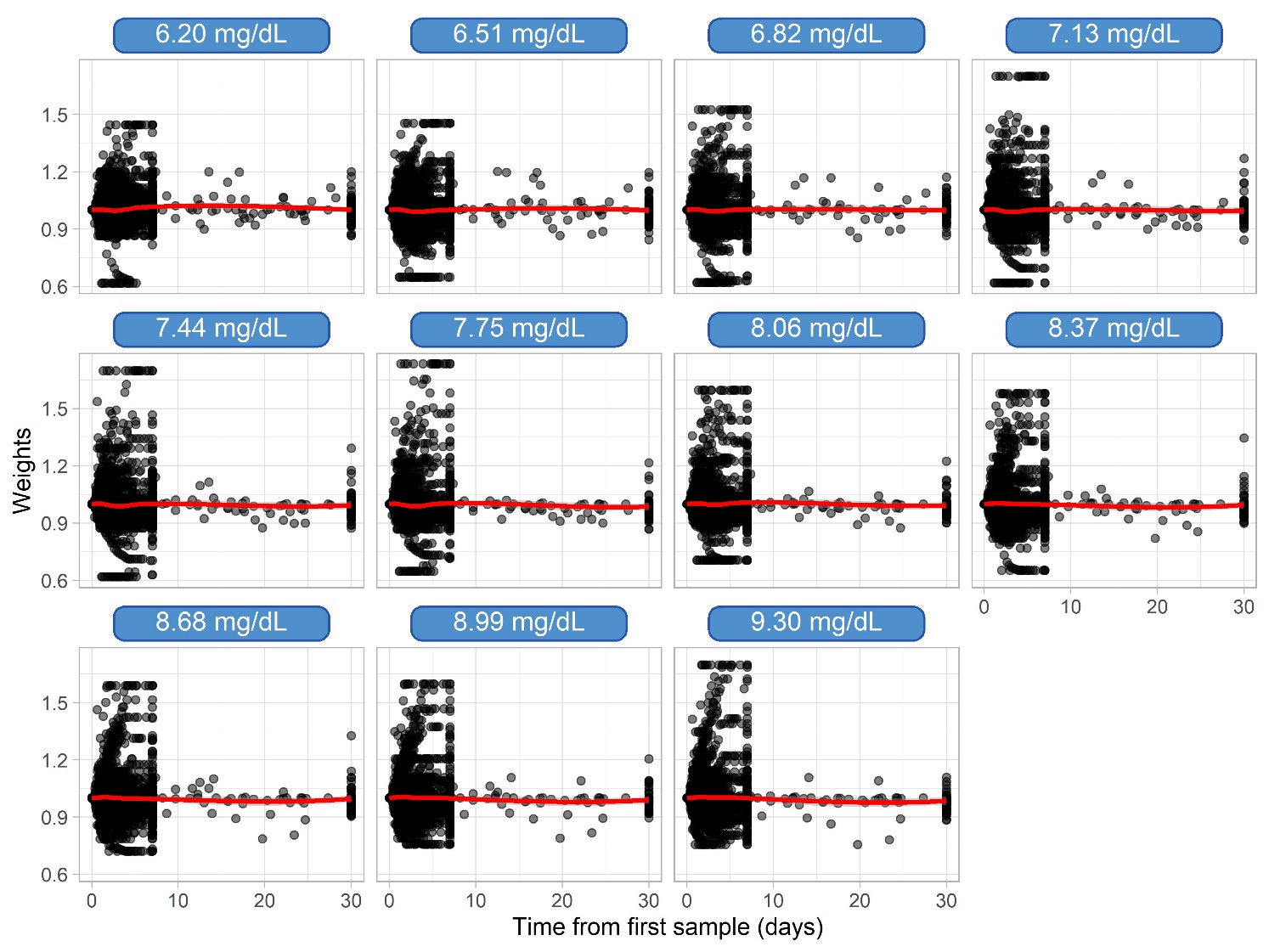


**Figure S2: Absolute standardized mean differences at 3 days after the first sample, between phosphatemia and conventional KRT strategy groups, according to the different thresholds of phosphatemia.**


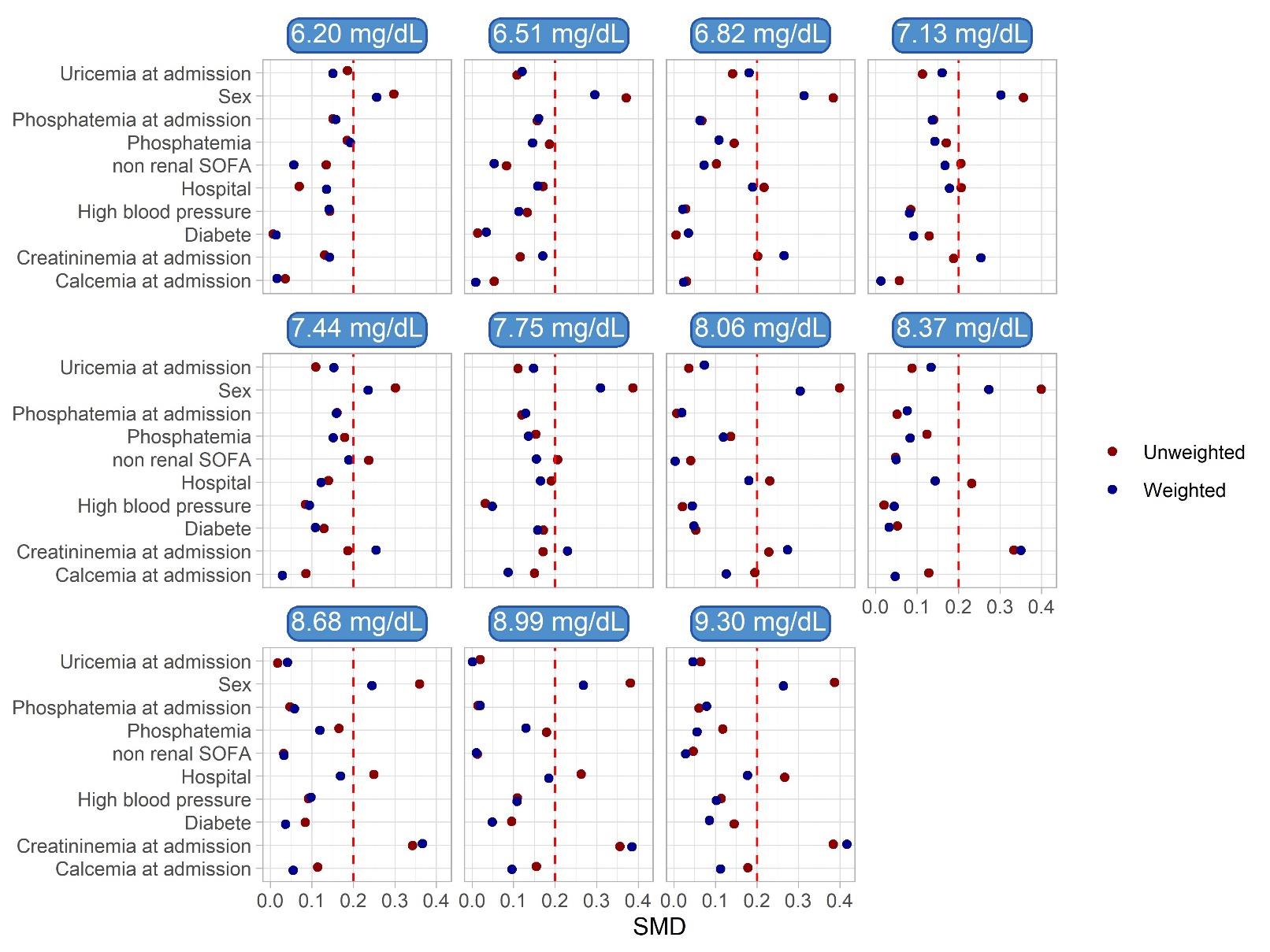


Sex and creatininemia at admission (SMD > 0.2) remained imbalanced. The analysis model was therefore adjusted on both variables.
