# Supplementary material for: Impact of early versus conventional kidney replacement therapy initiation in tumor lysis syndrome: a target trial emulation
Source: Ann Intensive Care. 2025 Apr 4;15:49. doi: 10.1186/s13613-025-01439-x (PMC11968619; doi:10.1186/s13613-025-01439-x)
Supplement: Supplementary file 3 — Supplementary Material 3 [file 13613_2025_1439_MOESM3_ESM.docx]

**Supplementary file 3: Sensitivity analyses**

**Figure S3: Hazard ratios (dots) and 95% confidence interval (horizontal segments) for the risk of MAKE 30 in phosphatemia strategy vs. conventional strategy, according to the phosphatemia** **threshold**. **Results with the addition of an interaction term between hospital (Saint Louis vs Angers) and SOFA in the censoring model**


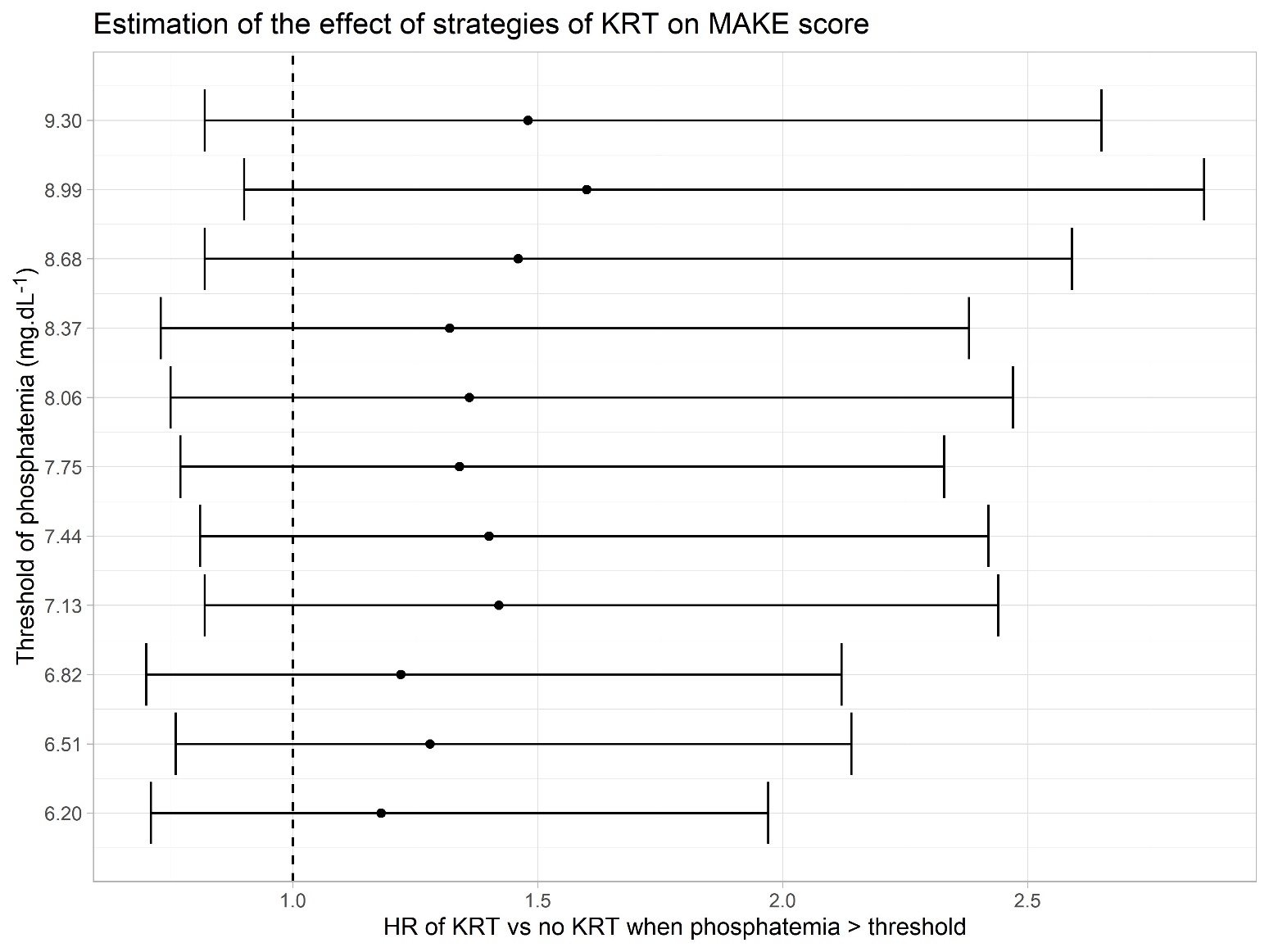


**Figure S4: Hazard ratios (dots) and 95% confidence interval (horizontal segments) for the risk of MAKE 30 in phosphatemia strategy vs. conventional strategy, according to the phosphatemia** **threshold**. **Results with the addition of interaction terms, between hospital (Saint Louis vs Angers) and SOFA and between hospital (Saint Louis vs Angers) and creatininemia at admission in the censoring model.**


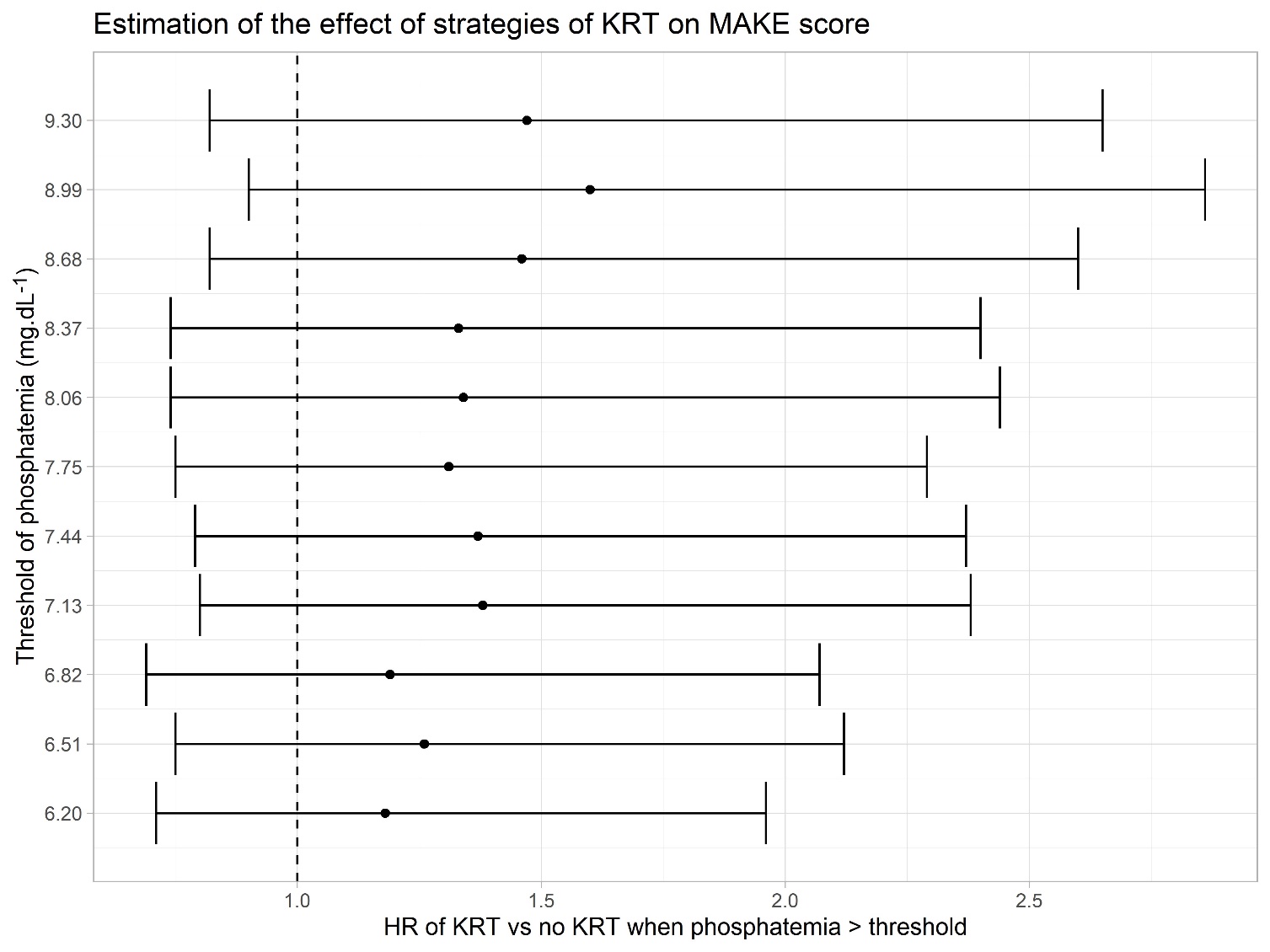


**Figure S5: Hazard ratios (dots) and 95% confidence interval (horizontal segments) for the risk of MAKE 30 in phosphatemia strategy vs. conventional strategy, according to the phosphatemia** **threshold. Results with untruncated weights.**


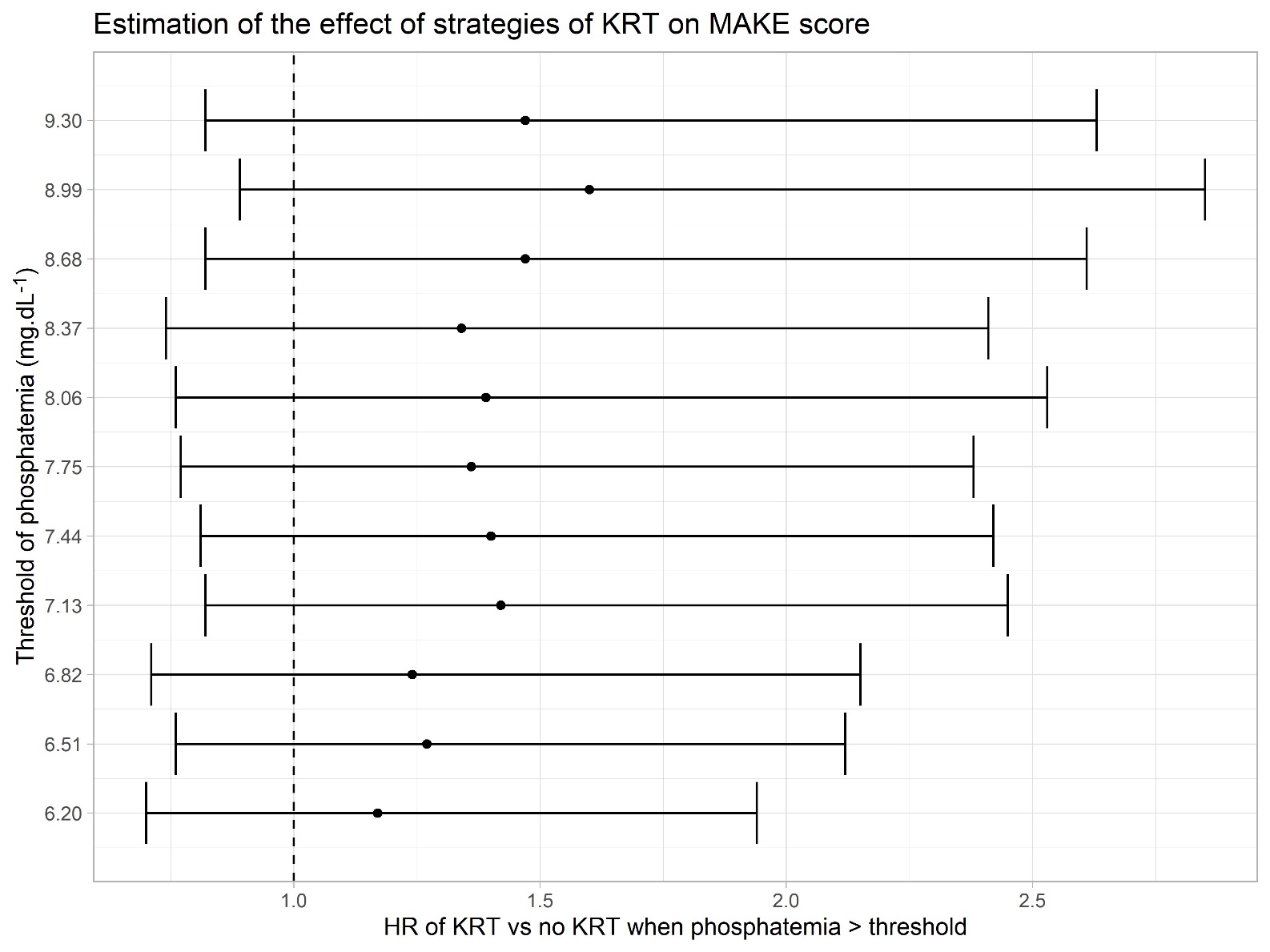


**Supplemental Table 1. Secondary analysis: standard univariate analysis of factors associated with MAKE 30 using logistic regression models.**

| **Variable** | **OR** | **P value** | **N** |
| --- | --- | --- | --- |
| Gender: Male | 1.23 [0.67;2.33] | 0.507 | 217 |
| Non hodgkin lymphoma | 1 (Reference) |  |  |
| Acute leukemia | 1.37 [0.74;2.51] | 0.312 | 217 |
| Other blood disorder | 2.65 [0.76;9.66] | 0.123 | 217 |
| Chronic renal failure | 0.55 [0.12;1.86] | 0.375 | 217 |
| HIV | 1.40 [0.62;3.09] | 0.408 | 217 |
| Hypertension | 1.89 [1.03;3.47] | 0.039 | 217 |
| Diabetes mellitus | 1.94 [0.87;4.30] | 0.099 | 217 |
| Chronic heart failure | 0.93 [0.28;2.72] | 0.896 | 215 |
| Clinical tumor lysis syndrome | 3.19 [1.69;6.31] | 0.001 | 217 |
| Non renal SOFA | 1.30 [1.19;1.43] | <0.001 | 217 |
| Log (day 1 diuresis in mL) | 0.74 [0.58;0.89] | 0.004 | 98 |
| Weight at admission in kg | 1.00 [0.99;1.02] | 0.607 | 195 |
| Potassium at admission in mmol/L | 0.90 [0.63;1.28] | 0.571 | 211 |
| Serum creatinine at admission in mg/dL | 1.01 [1.00;1.01] | <0.001 | 217 |
| Calcium at admission in mg/dl | 0.41 [0.16;0.94] | 0.048 | 210 |
| LDH at admission (by 1000) | 1.04 [0.97;1.11] | 0.256 | 198 |
| Disseminated intra-vascular coagulation | 2.29 [1.18;4.46] | 0.014 | 217 |
| No KRT at day 7 | 1 (Reference) |  |  |
| KRT during the first 7 days | 2.64 [1.46;4.86] | 0.002 | 214 |

HIV: Human Immunodeficiency Virus, LDH: Lactate DeHydrogenase, Modified SOFA: SOFA without kidney component, KRT: Kidney Replacement Therapy.
